# Treatment-Related Death during Concurrent Chemoradiotherapy for Locally Advanced Non-Small Cell Lung Cancer: A Meta-Analysis of Randomized Studies

**DOI:** 10.1371/journal.pone.0157455

**Published:** 2016-06-14

**Authors:** Jing Zhao, Yingfeng Xia, Joseph Kaminski, Zhonglin Hao, Frank Mott, Jeff Campbell, Ramses Sadek, Feng-Ming (Spring) Kong

**Affiliations:** 1 Department of Radiation Oncology, GRU Cancer Center/Medical College of Georgia, Georgia Regents University, Augusta, GA, United States of America; 2 Department of Oncology, Tongji Hospital, Tongji Medical College, Huazhong University of Science and Technology, Wuhan, China; 3 The First Hospital of Yichang, The People's Hospital of Three Gorges University, Yichang, China; 4 Department of Internal Medicine, GRU Cancer Center/Medical College of Georgia, Georgia Regents University, Augusta, GA, United States of America; 5 Department of Biostatistics and Epidemiology, GRU Cancer Center/Medical College of Georgia, Georgia Regents University, Augusta, GA, United States of America; Taipei Medical University, TAIWAN

## Abstract

Treatment related death (TRD) is the worst adverse event in chemotherapy and radiotherapy for patients with cancer, the reports for TRDs were sporadically. We aimed to study TRDs in non-small cell lung cancer (NSCLC) patients treated with concurrent chemoradiotherapy (CCRT), and determine whether high radiation dose and newer chemotherapy regimens were associated with the risk of TRD. Data from randomized clinical trials for locally advanced/unresectable NSCLC patients were analyzed. Eligible studies had to have at least one arm with CCRT. The primary endpoint was TRD. Pooled odds ratios (ORs) for TRDs were calculated. In this study, a total of fifty-three trials (8940 patients) were eligible. The pooled TRD rate (accounting for heterogeneity) was 1.44% for all patients. In 20 trials in which comparison of TRDs between CCRT and non-CCRT was possible, the OR (95% CI) of TRDs was 1.08 (0.70–1.66) (*P* = 0.71). Patients treated with third-generation chemotherapy and concurrent radiotherapy had an increase of TRDs compared to those with other regimens in CCRT (2.70% vs. 1.37%, OR = 1.50, 95% CI: 1.09–2.07, *P* = 0.008). No significant difference was found in TRDs between high (≥ 66 Gy) and low (< 66 Gy) radiation dose during CCRT (*P* = 0.605). Neither consolidation (*P* = 0.476) nor induction chemotherapy (*P* = 0.175) had significant effects with increased TRDs in this study. We concluded that CCRT is not significantly associated with the risk of TRD compared to non-CCRT. The third-generation chemotherapy regimens may be a risk factor with higher TRDs in CCRT, while high dose radiation is not significantly associated with more TRDs. This observation deserves further study.

## Introduction

Lung cancer remains the leading cause of cancer-related death worldwide [1]. As stated in the National Comprehensive Cancer Network (NCCN) guidelines [2], the standard treatment for locally advanced and unresectable non-small cell lung cancer (NSCLC) is concurrent administration of platinum-based chemotherapy regimens and thoracic external beam radiation. Recommendations for concurrent regimens include cisplatin/carboplatin with etoposide/vinblastine/pemetrexed/paclitaxel, and the definitive recommended radiation dose is 60–70 Gy in 2 Gy daily fractions.

Phase III randomized trials have demonstrated a survival advantage of concurrent chemoradiotherapy (CCRT) for locally advanced NSCLC patients compared to non-CCRT (sequential chemotherapy or radiotherapy or radiotherapy alone) [3, 4]. However, the 5-year overall survival rate remained at only 15% for those patients treated with CCRT [5]. Some recent studies indicated that local tumor control and survival would be further improved with more intensive therapy such as a high radiation dose to tumors through hyper- or hypofractionated delivery [3, 6, 7], or new regimens for concurrent chemotherapy [8, 9]. Moreover, consolidation chemotherapy after CCRT was also considered to improve therapeutic efficacy. Inevitably, treatment-related toxicities after CCRT can affect the quality of life and might even put patients at risk of death. The common causes of treatment-related death (TRD), including toxicities in lung, esophagus, and hematopoietic systems, have not been thoroughly analyzed partly due to sporadic occurrences in each trial.

In this study, we aim to 1) compare TRD rates among patients treated with CCRT or non-CCRT in randomized clinical trials, and 2) determine whether treatment factors such as high radiation dose and chemotherapy regimens during CCRT have an impact on TRD rates.

## Materials and Methods

This meta-analysis was performed according to the Preferred Reporting Items for Systematic Reviews and Meta-Analyses (PRISMA) statement (S1 Table) [10].

### Study Design, Search Strategy and Study Selection

Eligible trials included randomized, controlled studies with at least one CCRT arm for patients with locally advanced or unresectable NSCLC. All patients were chemotherapy/radiotherapy naïve prior to enrollment. TRD was defined as a fatal adverse event not attributable to tumor progression or other known etiologies, occurring within 30 days of the completion of treatment. TRD was reported by investigators as ‘possibly’, ‘probably’ or ‘definitely’ toxicity-related to treatments [11, 12].

Eligible trials were identified by searching electronic databases (PubMed, Cochrane and Embase) with a publication time before May 31, 2015, using the Cochrane Collaboration optimal search strategy. The keywords for literature searching included: non-small cell lung cancer, locally advanced, mortality/death/grade 5, chemoradiotherapy and randomized. This was supplemented by manual searches (reference lists of trial publications, review articles, relevant books, and meeting proceedings of the American Society of Clinical Oncology and International Association for the Study of Lung Cancer). Investigators and experts were also asked to help identify trials.

### Data collection

The data collected included age, gender, Zubrod score, smoking status, pathology type, weight loss before therapy, clinical stage, median overall survival, median progression-free survival, tumor response, and evaluation criteria after treatments. Total radiation dose and fractions in each trial were recorded. The chemotherapy data such as regimens and compliance were also collected. Causes of TRDs were recorded by each trial.

Two authors (JZ and YX) independently extracted data. All the data were checked for internal consistency and compared with the trial’s protocol and published reports. Data were checked for missing values, validity, and consistency across variables with the criteria in the Cochrane Handbook for Systematic Reviews of Interventions Version 5.1.0. The adequacy of the method of randomization was also assessed as described (by JZ and YX). Disagreements between review authors were resolved by discussion with the third author (JK).

### Statistical analysis

The primary endpoint for the study was TRD. To minimize potential bias due to heterogeneity, a pooled estimate for TRD was obtained with a Bayesian hierarchical model. Odds ratios (ORs) and 95% confidence interval (CI) were estimated for TRDs. χ^2^ tests and I^2^ were used to assess whether or not there was heterogeneity in TRD rates across the studies. If the test indicated heterogeneity across studies (*P* < 0.10 or I^2^ > 50%) [13], the random effects model (Der Simonian-Laird method) was selected. Otherwise, the fixed effects model (Mantel-Haenszel method) was used to distinguish between treatment groups. All hypothesis tests were two-sided, and *P* = 0.05 was considered to be significant. Publication bias was evaluated visually using funnel plots and statistically using Begg’s and Egger’s regression models, in which a *P*-value < 0.10 was considered statistically significant. RevMan version 5.2 and GraphPad Prism version 6.0 software were used for statistical analyses.

## Results

### Studies

**Fig 1:**
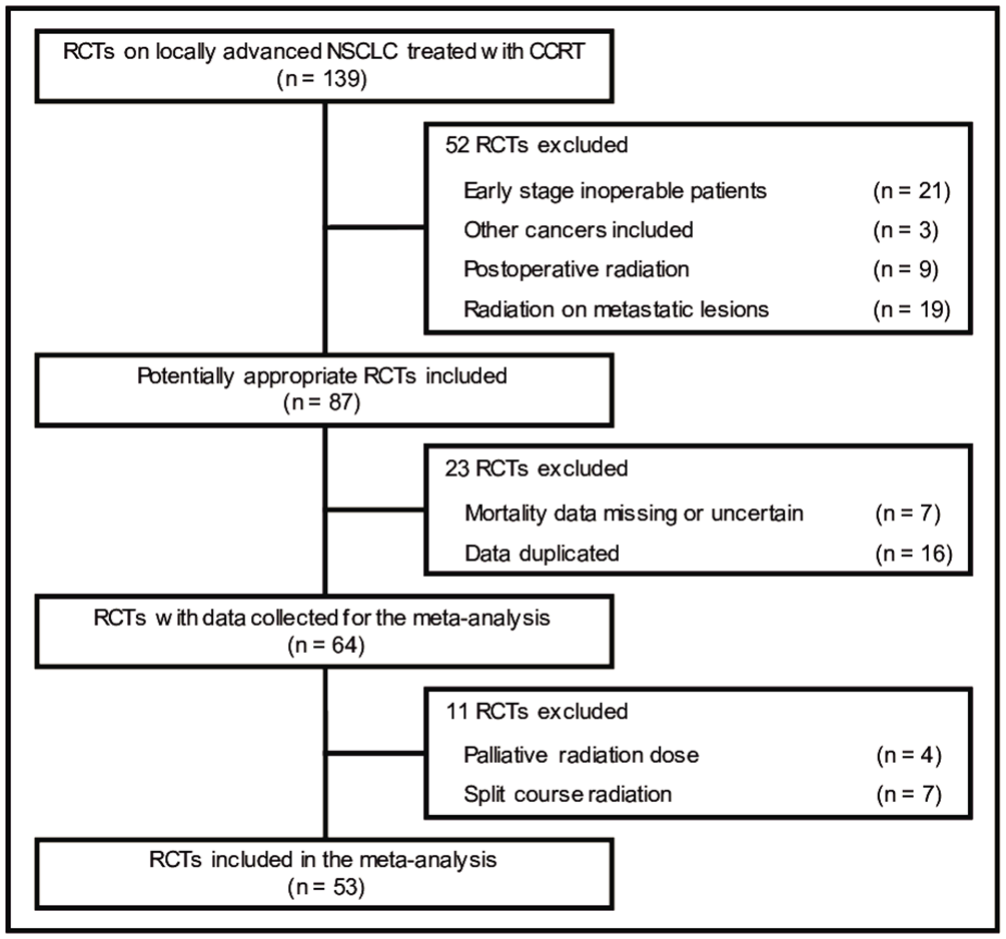
Flow chart of clinical trial selection. *Abbreviations*: RCTs, randomized clinical trials; NSCLC, non-small cell lung cancer; CCRT, concurrent chemoradiotherapy.

A total of 139 clinical trials were identified at the initial search of randomized trials including CCRT for locally advanced NSCLC. Eighty-six trials were excluded due to 1) TRD data missing, 2) duplications of publication or publications with overlap data, 3) treatments prior to CCRT, or 4) studies with mixed diagnoses such as small cell lung cancer. Finally, 53 trials with 8940 patients were eligible for this analysis (Fig 1). Each trial had one or more arms of a given factor such as radiation dose, chemotherapy regimens, induction chemotherapy and consolidation chemotherapy. The count of number of arms was based on factors under consideration. For example, a trial comparing induction chemotherapy versus non-induction chemotherapy with both arms using high dose radiation dose (≥ 66 Gy) were counted as two arms for high dose radiation. As a result, induction chemotherapy before CCRT and consolidation chemotherapy after CCRT were seen in 20 and 14 arms, respectively. Some newer chemotherapy regimens (3rd-generation) such as vinorelbine, paclitaxel, docetaxel, irinotecan, gemcitabine and pemetrexed were used in 46 arms, and 32 CCRT arms included treatment with high dose (≥ 66 Gy) radiotherapy. Patient characteristics before treatments were balanced between CCRT and non-CCRT arms (Table 1).

**Table 1:** Patient characteristics between CCRT and non-CCRT.

Patient characteristics	CCRT arm (n = 92)		non-CCRT arm (n = 25)		*P*
	Median	95% CI	Median	95% CI	
Age	61.0	60.59–63.16	61.0	59.34–64.50	0.98
Gender (male: female)	2.7	3.05–4.93	4.2	3.11–6.70	0.40
Pathology					
Adenocarcinoma (%)	32.7	27.66–36.56	30.0	24.87–35.23	0.56
Stage					
IIIa (%)	40.9	36.17–45.45	45.1	35.85–55.25	0.45
ECOG score					
0 (%)	47.6	40.26–52.94	44.3	32.87–58.30	0.89
Smoking status					
Smokers (%)	83.5	65.21–91.28	92.0	75.97–99.27	0.33
Weight loss					
≤ 5% (%)	67.2	41.12–68.83	32.6	18.87–52.77	0.78
Radiotherapy					
Dose (Gy)	60	60.21–63.43	60	58.53–63.07	0.40
Fraction	30	31.60–36.40	30	29.08–35.83	0.37

*Abbreviations*: CCRT, concurrent chemoradiotherapy; CI, confidence interval; ECOG, Eastern Cooperative Oncology Group.

### TRDs in all studies

In total, there were 214 TRDs. The most common cause of TRDs was radiation pneumonitis (accounting for 33.2% of TRDs). The second most common causes, with each accounting for more than 5% of TRDs, included neutropenia, pneumonia, hemorrhage, infection, acute respiratory distress syndrome (ARDS) and cardiac diseases (Table 2). The adjusted pooled TRD rate from all trials in this study was 1.44% (95% CI: 1.03–1.98%).

**Table 2:** Causes of TRDs in 53 trials.

Cause of TRDs	No. of TRDs (n = 214)	%
Radiation pneumonitis	71	33.2
Pneumonia	21	9.8
Hemorrhage	17	7.9
Pulmonary edema	1	0.5
ARDS	16	7.5
Pulmonary embolism	4	1.9
Infection	17	7.9
Neutropenia	33	15.4
Cardiac diseases	13	6.1
Esophagitis	3	1.4
Renal failure	4	1.9
Neuropathy	3	1.4
Others	11	5.1

*Abbreviations*: TRD, treatment related death; ARDs, Acute Respiratory Distress Syndrome.

### Comparison of TRD rate in trials with both CCRT and non-CCRT arms

**Fig 2:**
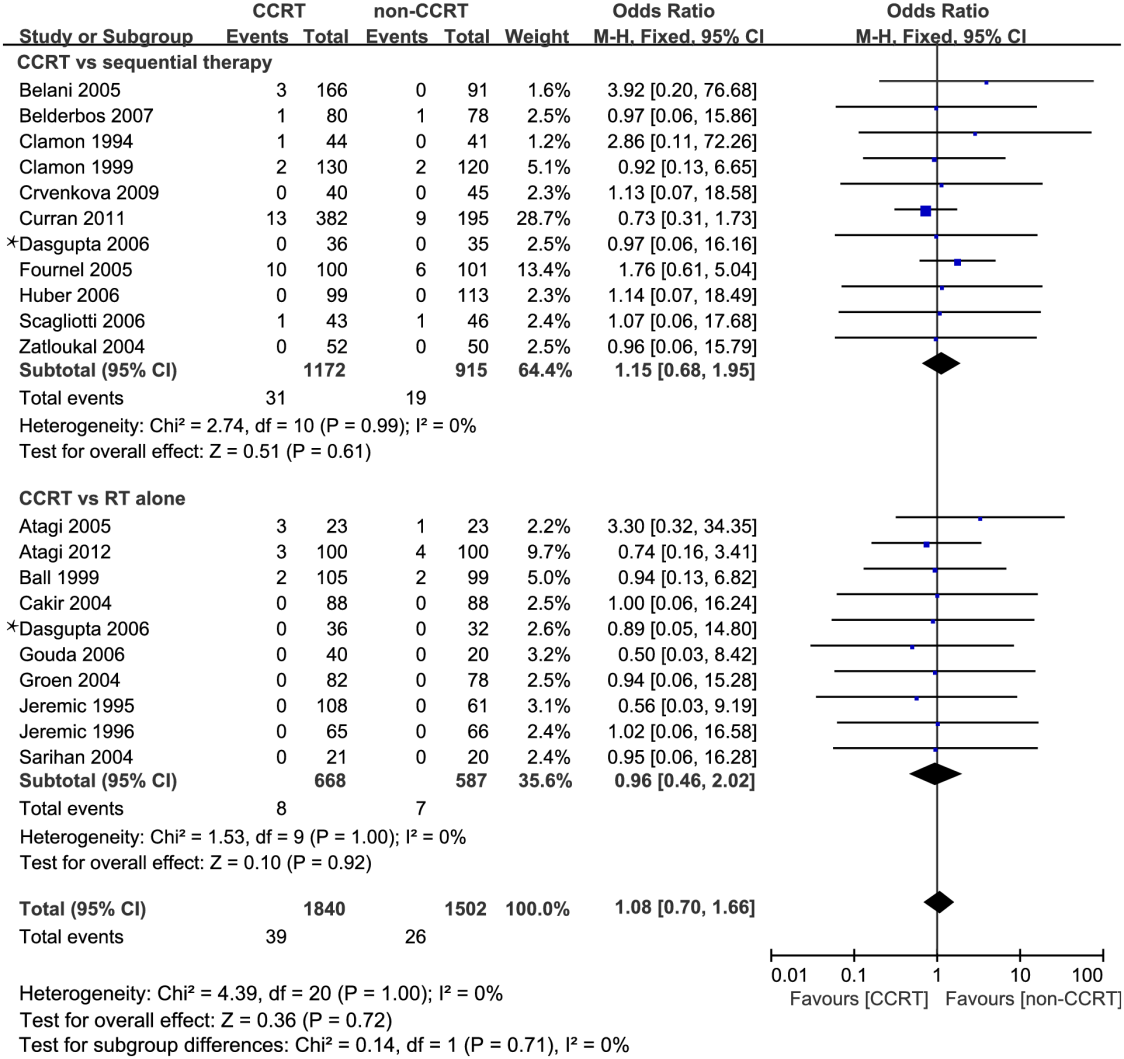
Comparison of TRDs in 20 studies with CCRT vs non-CCRT. CCRT data in Dasgupta’s study (*) were calculated twice as it included both arms of sequential chemoradiotherapy and RT alone. *Abbreviations*: TRD, treatment-related death; CCRT, concurrent chemoradiotherapy; RT, radiotherapy; CI, confidence interval.

Twenty trials [3, 4, 9, 14–30] (3306 patients) were included comparing TRDs between patients receiving CCRT and non-CCRT (S2 Table). A total of 65 TRDs (2.1%) was reported, including 39 patients in the CCRT group (2.16%) and 26 patients in the non-CCRT group (1.73%). The OR (95% CI) of TRDs for CCRT vs non-CCRT was 1.08 (0.70–1.66), *P* = 0.71. There was no evidence of statistically significant heterogeneity with an I^2^ value of 0% (χ^2^ test for heterogeneity, *P* = 1.00). The forest plot is shown in Fig 2. Begg’s funnel plots were used to assess the potential publication bias with log-transformed ORs calculated from TRDs (horizontal axis) as the outcome and their standard errors (SEs) (vertical axis) as the index for accuracy. The results showed that all the points in the funnel plots were symmetrically distributed with *P* values of 0.363, 0.754 and 0.858 for all the 20 studies and two subgroups, respectively, which indicated that there was no significant bias (S1 Fig).

**Fig 3:**
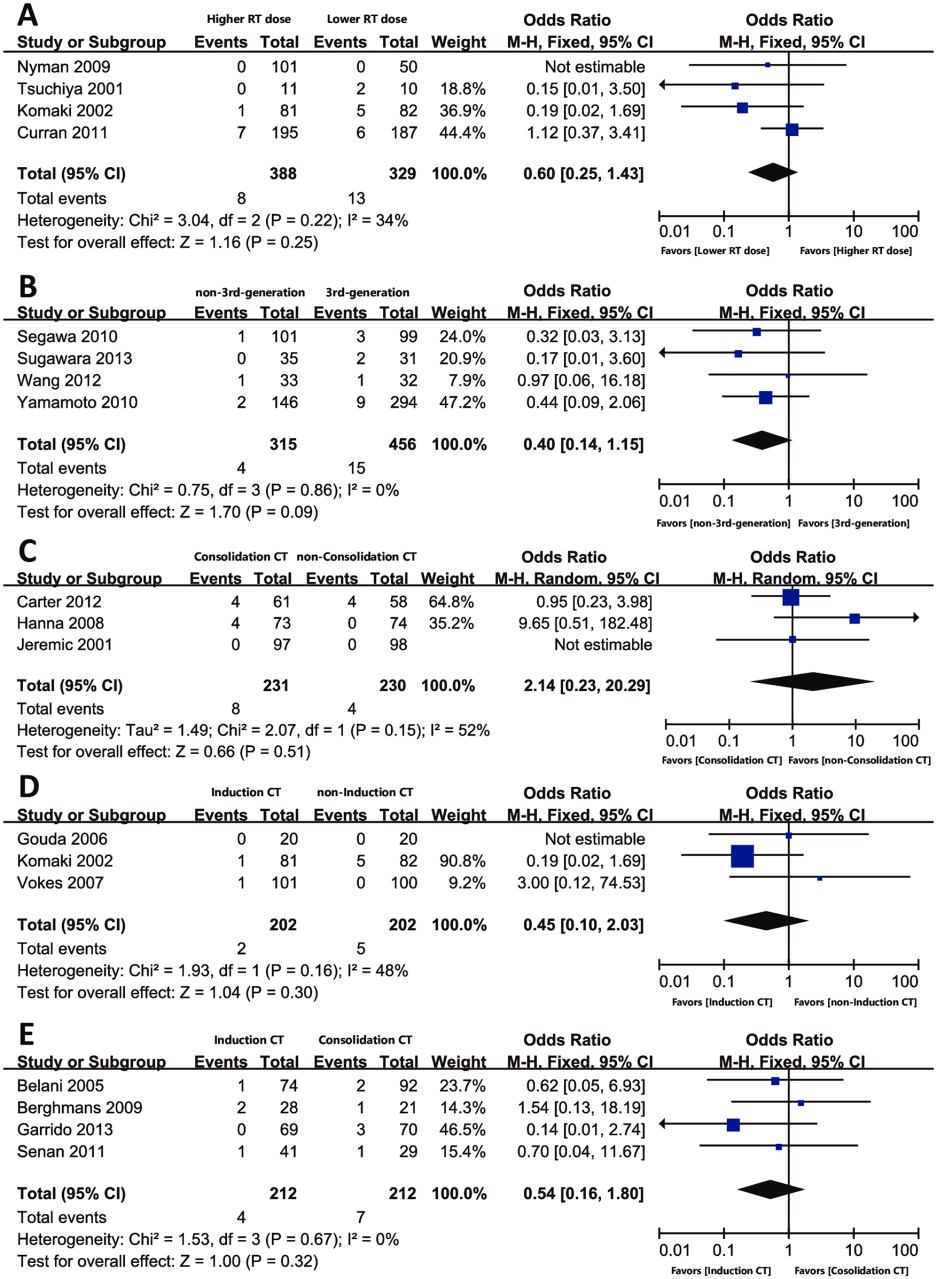
Risk factors associated with TRDs in randomized trials. Comparison of TRDs in CCRT trials with (A) higher vs. lower radiation dose, (B) newer vs. other chemotherapy regimens, (C) consolidation vs. non-consolidation chemotherapy after CCRT, (D) induction vs. non-induction chemotherapy before CCRT, and (E) induction vs. consolidation chemotherapy. *Abbreviations*: TRD, treatment-related death; RT, radiotherapy; CT, chemotherapy; CCRT, concurrent chemoradiotherapy; CI, confidence interval.

In 4 trials that included arms of both higher and lower radiation dose, the OR (95% CI) of TRDs was 0.60 (0.25–1.43), *P* = 0.25. There was no evidence of statistically significant heterogeneity with an I^2^ value of 34% (χ^2^ test for heterogeneity, *P* = 0.22) (Fig 3A). The newer chemotherapy regimens showed increased TRDs in CCRT compared to other regimens with a borderline significance (OR = 0.40, 95% CI: 0.14–1.15, *P* = 0.09) (Fig 3B). There are 3, 3 and 4 trials with arms of both CCRT and CCRT + consolidation chemotherapy, CCRT and induction chemotherapy + CCRT, and induction chemotherapy + CCRT and CCRT + consolidation chemotherapy, respectively. After pooling the data, no significance of TRDs was observed in either of the groups (Fig 3C–3E).

### Risk factors with TRDs in CCRT arms

**Fig 4:**
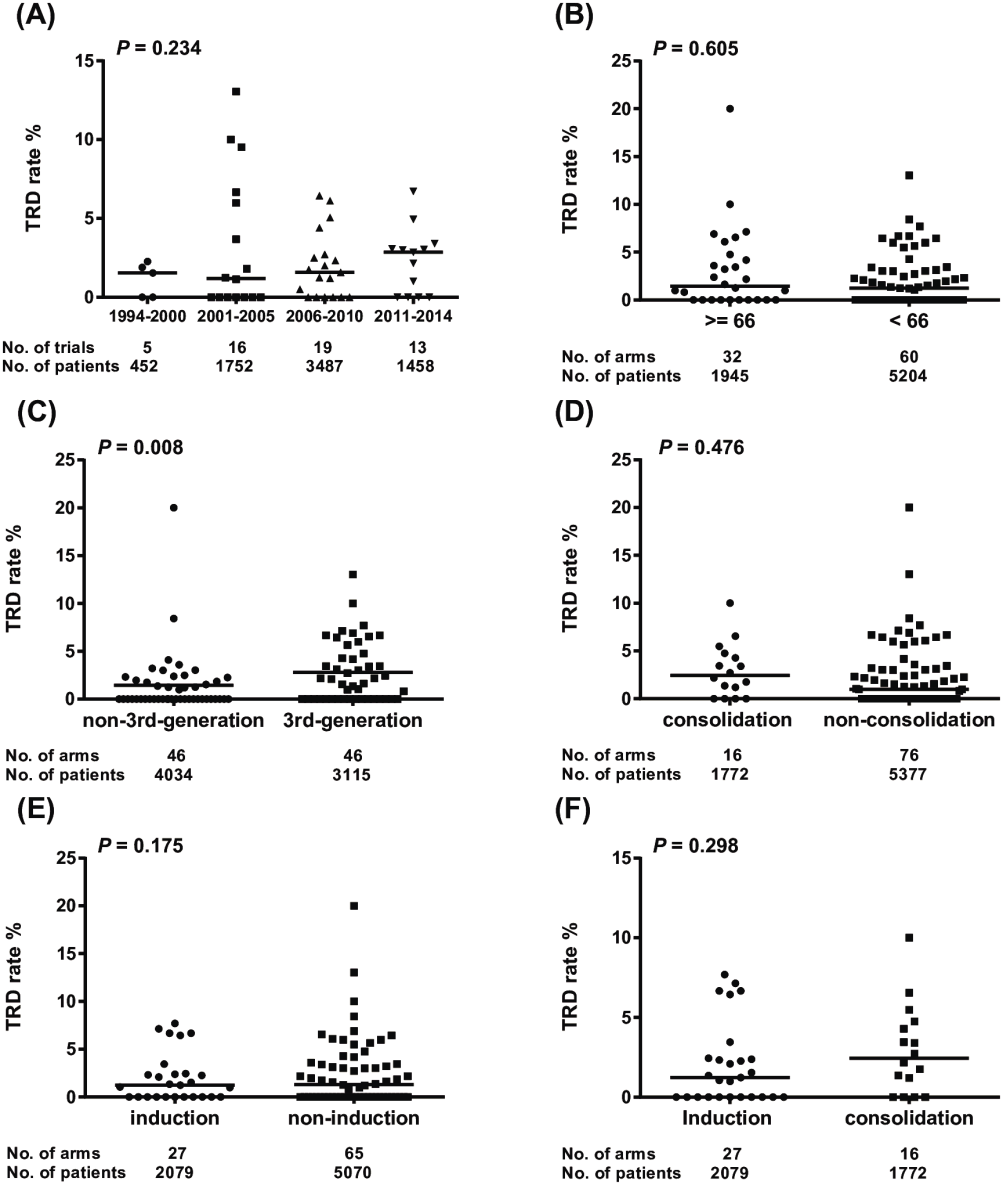
Distribution of risk factors and TRD rate in all CCRT arms. (A) TRD rates among trials with different study periods. (B) TRD rates between arms with high and low radiation dose. (C) TRD rates between arms with newer and other chemotherapy regimens. (D) TRD rates between arms with consolidation and non-consolidation chemotherapy after CCRT. (E) TRD rates between arms with induction and non-induction chemotherapy before CCRT. (F) TRD rates between arms with induction and consolidation chemotherapy. Number of arms was added and combined from all trials. For example, a trial comparing induction chemotherapy + CCRT vs. CCRT with both arms using high dose radiation dose (≥ 66 Gy) were counted as two arms for high dose radiation. The horizontal bar represents the median value. The *P* value was calculated by the log-rank test. *Abbreviations*: TRD, treatment-related death.

Further study for TRD-related factors were performed in the CCRT arms of all 53 trials. TRD rates among study periods were not significantly different shown, as in Fig 4A, *P* = 0.234. In Fig 4C, patients receiving newer (3rd-generation) regimen (46 arms, 3115 patients) with concurrent radiation had a significantly higher TRD rate compared with those treated with other regimens (46 arms, 4034 patients); the median TRD rates were 2.70% vs. 1.37%, OR = 1.50, 95% CI: 1.09–2.07, *P* = 0.008. There was no significant difference in TRD rates between high radiation dose (≥ 66 Gy, 32 CCRT arms, 1945 patients) than those with low radiation dose (< 66 Gy, 60 CCRT arms, 5204 patients) as shown in Fig 4B (median TRD rate, 1.31% vs 1.06%, OR = 0.92, 95% CI: 0.66–1.28, *P* = 0.605). Similarly, neither consolidation after chemotherapy (*P* = 0.476) nor induction before chemotherapy (*P* = 0.175) had a significant effect with increased TRDs in this study (Fig 4D and 4E).

A mixed effect linear model was used to assess if study period, radiation dose, chemotherapy regimens, consolidation chemotherapy after CCRT and induction chemotherapy before CCRT had a significant effect on the TRD rate. As a result, neither of the factors was significantly correlated with TRD rate, with *P* values of 0.61, 0.64, 0.37, 0.81, and 0.19, respectively.

## Discussion

In this study, we identified 214 TRDs in 53 prospective randomized trials. Compared to sequential chemoradiotherapy and radiotherapy alone, CCRT was not significantly correlated with more TRDs. Higher radiation dose, consolidation chemotherapy after CCRT or induction chemotherapy before CCRT did not significantly increase the risk of TRD. Newer regimens in a concurrent course significantly increased TRDs compared to other regimens. To our knowledge, this study comprehensively summarized TRDs after radiation-based treatment in NSCLC patients for the first time. The TRD data are critically important for physicians and patients for decision making of treatment options.

TRD is the worst possible adverse event and should be avoided. The fact that CCRT does not increase the risk of TRD may encourage physicians to use CCRT more often, particularly for traditional physicians who are often concerned about TRD or treatment toxicities from CCRT. We found in our study that the overall rate of TRD associated with CCRT was 1.44%. However, it has been reported that radiation pneumonitis could cause the TRD rate to be as high as 10% after CCRT [31]. Unfortunately, the risk factors with TRDs remain poorly understood. Ohe’s study [32] indicated that advanced age, poor performance status, pulmonary function and elevated LDH level were significant factors with high risk of TRDs. Minami-Shimmyo [33] reported that concurrent therapy of gefitinib was associated with more TRDs because of higher morbidity of interstitial lung disease (ILD). Moreover, radiation dosimetric factors could also be correlated with TRDs. According to Song et al [34], the percentage of total lung volume receiving a radiation dose of 5 Gy (total lung V_5_), contralateral lung V_5_ and V_10_ were the main determinants of high risk of TRDs, in which contralateral lung V_5_ was the independent predictor for TRD. Although a limited number of events from this study did not provide us adequate power to perform any meaningful analysis, we should pay extreme caution to patients with the high risk factors that have be previously reported, such as ILD [35], and minimize the radiation dose to normal lung tissue.

The radiation dose effect on overall survival (OS) is controversial. High dose radiation has been demonstrated to be advantageous to survival. In a study from Michigan [36], the 5-year OS rate was 28% in the 92–103 Gy group, which is significantly higher than that in the 63–69 Gy group (4%). This suggests that high dose radiation could be a promising modality if delivered under strict dose limitations for organs at risk (OARs). A secondary analysis of RTOG (Radiation Therapy Oncology Group) studies using CCRT demonstrated the survival benefits of high dose radiotherapy, showing a 3% reduction of death risk with each Gy of dose escalation in patients treated in the range of 60–70 Gy in RTOG trials [37]. However, high dose is often considered to be a risk factor for TRDs, and unexpected toxicities of OARs often limit its widespread use. In JCOG 8902 [38], only 30% of patients could tolerate 60 Gy and complete radiotherapy in concurrent course. In the RTOG 0617 trial, a significant increased risk of deaths were found in the high dose arms (median survival 19.5 months (74 Gy arm) vs 28.7 months (60 Gy arm), *P* = 0.0007), and there were more TRDs in the high dose arm (10 vs 2 cases) [39]. This meta-analysis, including a dose range of 40–74 Gy, showed no significant differences between conventional and higher dose groups. Until data from a prospective study is available, the higher radiation dose (≥ 74 Gy) should be performed under a clinical trial setting with CCRT.

It is interesting to note that newer chemotherapy significantly increased the risk of TRD in this study. Platinum-based newer regimens were recognized to have better survival than first-line therapy for locally advanced or metastatic NSCLC patients; these regimens, however, have more hematological and gastrointestinal toxicities [40]. Taking this into consideration, newer chemotherapy regimens were not usually used at full doses in the concurrent phase. Several non-randomized studies, however, have shown their feasibility and safety. In Mornex’s one-arm study [41], no acute or late grade 3 or 4 toxicities were found after a full dose of pemetrexed (500 mg/m^2^) with CCRT in nine patients. Patients were treated with irinotecan (60mg/m^2^ per week) as concurrent chemotherapy in the JCOG 9706 [42] study. One TRD was observed in 68 patients due to radiation pneumonitis. A Cochrane review [43] including five trials assessing concurrent versus sequential therapy with full dose newer chemotherapy (docetaxel or paclitaxel) indicated that there were slightly higher TRDs (4% vs. 2%) with concurrent therapy, but this did not reach statistical significance (*P* = 0.088). The results of a randomized Phase III study comparing concurrent radiation with full dose cisplatin/pemetrexed or cisplatin/etoposide (the PROCLAIM trial) are expected [44]. More randomized trials are needed to demonstrate the safety of using full doses of newer chemotherapy regimens in a concurrent period.

This study may not be powered to determine whether consolidation chemotherapy after CCRT and induction chemotherapy before CCRT increased TRDs. Several clinical trials such as the HOG LUN 01–24 study [45], LAMP study [9] and CALGB 39801 study [46] have shown that consolidation/induction chemotherapy was associated with more toxicities, although better survival outcomes were obtained. However, due to the heterogeneity of the patient population in the studies, we have no strong evidence thus far that CCRT with extra chemotherapy is superior to CCRT alone. Thus, consolidation or induction chemotherapy should be cautiously applied, and clinical factors and prior therapies should be considered before decisions are made.

This study had some limitations. For example, TRD may also be associated with individual patient factors such as their baseline condition and responses to treatment, which could not be assessed in this meta-analysis. For more aggressive radiation, the trials of higher dose radiation (≥ 66 Gy) were limited in the numbers of studies, patients and dose levels (the highest dose was 74 Gy in all trials). This analysis was also limited for study power to detect small differences for consolidation chemotherapy and various choices of newer agents (i.e., which one was more toxic). Additionally, this study was not designed to detect the deaths associated with treatment that were not classified as TRDs; no significant difference in TRD is not equal to no harm or benefit. Nevertheless, this is the first study that compiled TRDs between CCRT and non-CCRT from randomized trials. The toxicities and benefit of full-dose newer chemotherapy with concurrent chemotherapy are needed to be validated in prospective randomized trials.

## Conclusions

TRDs associated with CCRT were comparable to those associated with sequential chemoradiotherapy or radiotherapy alone. The newer chemotherapy regimens may significantly increase TRDs with concurrent radiation. The application of high radiation dose (up to 74 Gy), consolidation chemotherapy after CCRT or induction chemotherapy before CCRT may not increase the risk of reported TRDs. Further studies with larger populations are expected to make more powerful determinations.

## Supporting Information

S1 Fig Begg’s funnel plots evaluating potential publication bias. The funnel shape distribution indicates no publication bias. *Abbreviations*: CCRT, concurrent chemoradiotherapy; OR, odds ratio; SE, standard error. Supporting information: The datasheet of 86 excluded studies during the data selction.
(TIF)

S1 Table PRISMA 2009 checklist. (DOC)

S2 Table Characteristics of 20 trials including both CCRT and non-CCRT arms. *Abbreviations*: CCRT, concurrent chemoradiotherapy; CT, chemotherapy; RT, radiotherapy.
(DOCX)
