# Supplementary figures and images for: Treatment-Related Death during Concurrent Chemoradiotherapy for Locally Advanced Non-Small Cell Lung Cancer: A Meta-Analysis of Randomized Studies
Source: PLoS One. 2016 Jun 14;11(6):e0157455. doi: 10.1371/journal.pone.0157455 (PMC4907424; doi:10.1371/journal.pone.0157455)

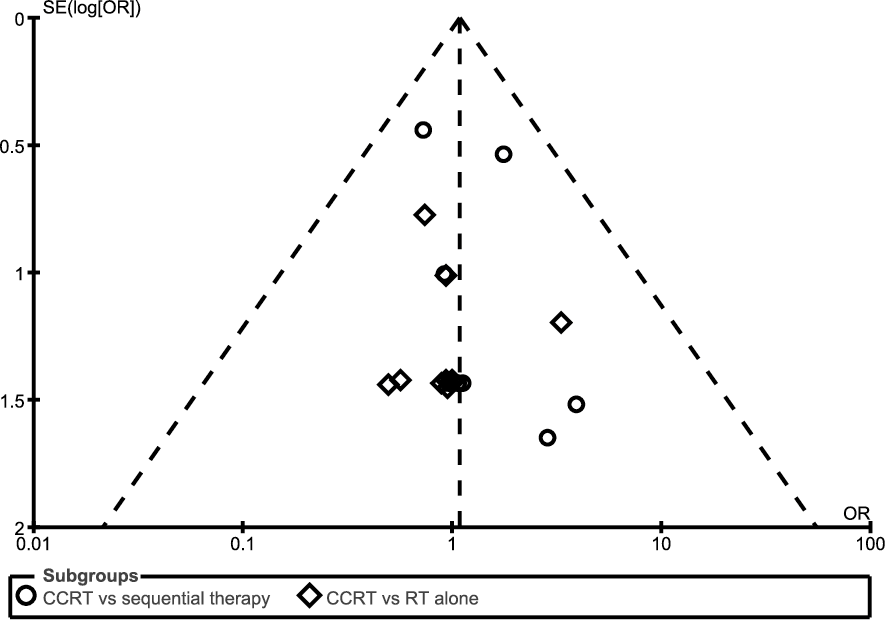

Supplement: S1 Fig — The funnel shape distribution indicates no publication bias. Abbreviations: CCRT, concurrent chemoradiotherapy; OR, odds ratio; SE, standard error. Supporting information: The datasheet of 86 excluded studies during the data selction. (TIF) [file pone.0157455.s001.tif]
