# Supplementary material for: Treatment-Related Death during Concurrent Chemoradiotherapy for Locally Advanced Non-Small Cell Lung Cancer: A Meta-Analysis of Randomized Studies
Source: PLoS One. 2016 Jun 14;11(6):e0157455. doi: 10.1371/journal.pone.0157455 (PMC4907424; doi:10.1371/journal.pone.0157455)
Supplement: S2 Table — Abbreviations: CCRT, concurrent chemoradiotherapy; CT, chemotherapy; RT, radiotherapy. (DOCX) [file pone.0157455.s003.docx]

S1 Table. Characteristics of 20 trials including both CCRT and non-CCRT arms.

| 1st author / Publication | Accrual period | No. of patients | CCRT group | | non-CCRT group | |
| --- | --- | --- | --- | --- | --- | --- |
|  |  |  | Concurrent CT | RT | CT (before RT) | RT |
| Clamon 1994 | 1988-1989 | 85 | Carboplatin 100 mg/m^2^/wk, wk1-6 | 60 Gy/30f, 6 wks | Vinblastine 5 mg/m^2^/wk, wk1-5; cisplatin 100 mg/m^2^, d1, 29 | 3 wks after CT, 60 Gy/30f, 6 wks |
| Clamon 1999 | 1991-1999 | 250 | Carboplatin 100 mg/m^2^/wk, wk1-6 | 60 Gy/30f, 6 wks | Vinblastine 5 mg/m^2^, d1, 8, 15, 22, 29; cisplatin 100 mg/m2, d1, 29 | 5 wks after CT, 60 Gy/30f, 6 wks |
| Zatloukal 2004 | 1997-2001 | 102 | Vinorelbine 12.5 mg/m^2^, d1, 8, 15, 29, 36, 43; cisplatin 80 mg/m^2^. d1,29 | 60 Gy/30f, 6 wks | Vinorelbine 12.5 mg/m^2^, d1, 8, 15, 29, 36, 43; cisplatin 80 mg/m^2^, d1,29 | 2 wks after CT, 60 Gy/30f, 6 wks |
| Belani 2005 | 1998-2001 | 257 | Paclitaxel 45 mg/m^2^/wk, carboplatin AUC = 2 mg/ml*min/wk, wk1-7 | 63 Gy/34f, 7 wks | Paclitaxel 200 mg/m^2^, carboplatin AUC = 6 mg/ml*min. d1, 22 | 3 wks after CT, 63 Gy/34f, 7 wks |
| Fournel 2005 | 1996-2000 | 201 | Etoposide 50 mg/m^2^, cisplatin 20 mg/m^2^, d1-5, 29-33 | 66 Gy/33f, 6.5 wks | Vinorelbine 30 mg/m^2^/wk, wk1-8; cisplatin 120 mg/m^2^, d1, 29 | 4 wks after CT, 66 Gy/33f, 6.5 wks |
| Dasgupta 2006 | Not stated | 103 | Etoposide 50 mg/m^2^, cisplatin 20 mg/m^2^, d1-5, d22-26 | 50 Gy/25f, 5 wks | 1) None  2) Etoposide 120 mg/m^2^, d1-3, 22-24, 43-45; cisplatin 80 mg/m^2^, d1, 22, 43 | 1) 65 Gy/30f, 6 wks  2) 3-4 wks after CT, 60 Gy/30f, 6 wks |
| Huber 2006 | 1997-2002 | 212 | Paclitaxel 60 mg/m^2^/wk, wk1-6 | 60 Gy/30f, 6 wks | Paclitaxel 200 mg/m^2^, carboplatin AUC = 6 mg/ml*min. d1, 22 | 3 wks after CT, 60 Gy/30f, 6 wks |
| Scagliotti 2006 | 1999-2001 | 89 | Docetaxel 20 mg/m^2^/wk, wk1-6 | 60 Gy/30f, 6 wks | Docetaxel 85 mg/m^2^, d1, 22; cisplatin 40 mg/m^2^, d1-2, 22-23 | 6 wks after CT, 60 Gy/30f, 6 wks |
| Belderbos 2007 | 1999-2003 | 158 | Cisplatin 6 mg/m^2^/d, wk1-5 | 66 Gy/24f, 5 wks | Gemcitabine 1250 mg/m^2^, d 1, 8, 22, 29; cisplatin 75 mg/m^2^, d2, 23 | 3 wks after CT, 66 Gy/24f, 5 wks |
| Crvenkova 2009 | 2005-2008 | 40 | Etoposide 100 mg/m^2^, cisplatin 30 mg/m^2^, d1-3, 29-31 | 60 Gy/30f, 6 wks | Etoposide 100 mg/m^2^, carboplatin AUC = 6 mg/ml*min. d1-3, 22-24, 43-45, 64-66 | 4 wks after CT, 60 Gy/30f, 6 wks |
| Curran 2011 | 1994-1998 | 577 | 1) Vinblastine 5 mg/m^2^/wk, w1-5; cisplatin 100 mg/m^2^, d1,29  2) etoposide 50 mg bid, wk1-6; cisplatin 50 mg/m^2^, d1, 8, 29, 36 | 1) 63 Gy/34f, 7 wks  2) 69.6Gy/58f, 6 wks | vinblastine 5 mg/m^2^/wk, wk1-5; cisplatin 100 mg/m^2^, d1,29 | 3 wks after CT,63 Gy/34f, 7 wks |
| Jeremic 1995 | 1988-1989 | 169 | 1) Etoposide 100 mg, d1-3; carboplatin 100 mg, d1-2, wk1-6  2) etoposide 100 mg, d1-5; carboplatin 200 mg, d1-2. wk1, 3, 5 | 64.8 Gy/54f, 5.5 wks | None | 64.8 Gy/54f, 5.5 wks |
| Jeremic 1996 | 1990-1991 | 131 | Etoposide 50 mg/d, carboplatin 50 mg/d, wk1-6 | 69.6 Gy/ 58f, 6 wks | None | 69.6 Gy/58f, 6 wks |
| Ball 1999 | 1989-1995 | 204 | Carboplatin 70 mg/m^2^, d1, 5, 29, 33 | 1) 60 Gy/ 30f, 6 wks  2) 60 Gy/ 30f, 3 wks | None | 1) 60 Gy/30f, 6 wks  2) 60 Gy/30f, 3 wks |
| Cakir 2004 | 1997-1999 | 176 | Cisplatin 20 mg/m^2^, d1-5, wk2, 6 | 64 Gy/32f, 6.5 wks | None | 64 Gy/30f, 6.5 wks |
| Groen 2004 | 1995-1998 | 160 | Carboplatin 20 mg/m^2^/d, wk1-6 | 60 Gy/30f, 6 wks | None | 60 Gy/30f, 6 wks |
| Sarihan 2004 | 1996-2001 | 41 | Paclitaxel 30-60 mg/m^2^/wk, wk1-6 | 59.4Gy/ 33f, 5.5 wks | None | 63 Gy/35f, 7 wks |
| Atagi 2005 | 1999-2001 | 46 | Carboplatin 30 mg/d, wk1-4 | 60 Gy/30f, 6 wks | None | 60 Gy/30f, 6 wks |
| Gouda 2006 | 1998-2000 | 60 | Paclitaxel 45 mg/m^2^/wk, carboplatin AUC = 2 mg/ml*min/wk, wk1-6 | 60 Gy/30f, 6 wks | None | 60 Gy/30f, 6 wks |
| Atagi 2012 | 2003-2010 | 200 | Carboplatin 30 mg/d, wk1-4 | 60 Gy/30f, 6 wks | None | 60 Gy/30f, 6 wks |

*Abbreviations:* CCRT, concurrent chemoradiotherapy; CT, chemotherapy; RT, radiotherapy.
